# From population to public institutions: what needs to be changed to benefit from the full value of vaccination

**DOI:** 10.3402/jmahp.v3.26965

**Published:** 2015-08-12

**Authors:** Thomas Szucs, Sibilia Quilici, Marina Panfilo

**Affiliations:** 1 European Center of Pharmaceutical Medicine, Institute of Pharmaceutical Medicine, University of Basel, Basel, Switzerland; 2 Sanofi Pasteur MSD, Lyon, France; 3 Sanofi Pasteur MSD, Roma, Italy

**Keywords:** vaccination, outbreaks, value, vaccine industry, economic evaluation, vaccines complexity

## Abstract

The poor perception of the benefits of vaccines, and their subsequent underuse, can result in substantial economic, societal, and political burden. Adequate support and communication from health authorities and governments is essential to promote the benefits of vaccination and reduce the risk of infectious diseases outbreaks. Cost-containment policies in the vaccine procurement processes could also be a threat to the long-term sustainability of the vaccine industry and manufacturing sites in Europe. Biologicals, such as vaccines, are highly technical and complex products to manufacture and only a few industries are engaged in this activity. Developing incentives to encourage vaccine manufacturers and identifying means of taking into consideration the specificities of vaccines in economic evaluations could allow the full value of vaccination to be appreciated. In conclusion, governments, international agencies, and other stakeholders have an important role to play to help society regain confidence in vaccination and ensure that the benefits of vaccination programmes are fully recognised and valued.

Vaccination has been shown to be a valuable intervention from medical, economic, and societal points of views. It is one of the rare interventions that has had such an important cross-sectorial contribution and population level impact. Despite this, benefits from vaccines are often poorly appreciated by the public and by governments leading to low vaccination coverage rates and reflecting a ‘societal disinvestment’ in vaccination programmes. In addition, the economic value of vaccines is often underestimated compared with that for curative drugs, as seen by the fact that the worldwide expenditure for vaccines represents only about 3% of the global medicines market (2010 figures) (1); in Italy and France, vaccines represented only 1.2 and 1.8% of the total pharmaceutical public expenditure in 2013 (2, 3).

There has been a substantial increase in healthcare expenses due to the rise of chronic diseases in populations. It is estimated that approximately 75% of Europe's healthcare budget is spent on chronic diseases, accounting for €700 billion annually (4). Although pharmaceutical companies have invested heavily in developing new therapies, very few have invested in the less attractive business potential of vaccines (high degree of complexity, low return on investment, etc.). Consequently, in the United States since 1967, the number of vaccine manufacturers has fallen from 37 to 10. Currently, 80% of the vaccine market is held by five manufacturers (5) with about $750 million spent on research and development compared with $26.4 billion for pharmaceuticals (6, 7). The current lack of recognition of the value of vaccination, increasing anti-vaccine lobbies, and price-driven vaccine purchasing processes (which do not acknowledge the specificity, complexity, and necessary investment for the development of vaccines) can have a short-, medium-, and long-term impact on the sustainability of the vaccine industry in Europe, with important health and economic consequences. This article addresses the need for a change in the perception of vaccines and recognition of their value to maximise their contribution to the promotion of healthier European populations.

## Suboptimal vaccine coverage

Over the past few decades, vaccination has led to the control and even elimination of several vaccine-preventable diseases in Europe. However, outbreaks of preventable diseases, such as measles or pertussis, continue to occur even in countries with well-established vaccination programmes. These outbreaks happen when the level of vaccination coverage in a population is not sufficiently high to contain the pathogen (8). First, this can occur when the population stops adhering to vaccination programmes because individuals do not fear a disease they do not know anymore. Thus, vaccines are ‘victims of their own success’ and outbreaks are due to the failure to vaccinate the susceptible population (9). Second, as for any biological product, adverse reactions, although extremely rare (1 per million doses administered), can have negative effects on public trust in vaccination, thus fear of the risk of the vaccine is greater than the fear of having the disease (10). Third, difference in vaccines type (i.e., live attenuated vs. inactivated vaccines), trade-off between efficacy and safety (i.e., whole cell vs. acellular), and the different capacity of individuals to respond efficiently to vaccines (i.e., decreased immunogenicity in the elderly) means that vaccines are not 100% efficacious and their duration of protection varies. This is why there are booster strategies for some vaccines in national vaccination programmes. For example, the increased incidence of pertussis, despite high vaccination coverage in infants, maybe due to several reasons, including increased diagnostic testing due to higher disease awareness and improved laboratory diagnostics and surveillance (11). Furthermore, neither natural infection nor vaccination provides lifelong immunity against pertussis, thus vaccination strategies to vaccinate almost exclusively children may not be sufficient and should be accompanied by booster vaccination in adolescents and adults (11, 12). The limited duration or level of protection that exists for some vaccines reinforces the need to achieve high coverage rates to maximise the direct and indirect effects of vaccination within the population.

Erosion of parents’ trust in vaccines is also linked to the many controversies and scares that have been brought to the public attention by the media and kept alive by anti-vaccine lobbies (13, 14). The pertussis vaccine controversy that started in the mid-1970s in the United Kingdom after the publication of a report alleging that 36 children suffered serious neurological conditions following DTP vaccination, is often considered as the re-activation of anti-vaccination opposition in modern days. This report attracted much media attention and waves of public concerns and, by 1977, vaccination coverage had declined from 77 to 33%. This was followed by three major epidemics of pertussis with over 100,000 cases and the deaths of at least 36 children. Nearly 25 years after the DTP controversy, the United Kingdom faced another major public crisis in vaccine confidence, due to an alleged link between measles-mumps-rubella (MMR) vaccination and autism (15). Measles vaccination rates in children fell from over 90% in 1997 to less than 80% in 2004 (13). Lastly, poorly managed vaccination campaigns in some countries have also led to widespread mistrust of vaccines and government vaccination programmes (16, 17).

In many countries in the WHO European Region where vaccination uptake has decreased over recent years, there has been a rise in the number of measles cases, with over 90,000 cases of measles in the past 3 years (Table 1) (18). In their recent initiative, WHO Europe reaffirmed the economic burden of these preventable diseases (19):

- A measles outbreak in Duisburg, Germany, in 2006 led to 311 schoolchildren missing a total of 2,854 school days, and 30 working adults missing 301 work days. The average cost of a measles case in Germany was estimated to be €520.
- A study in 10 Western European countries revealed that mothers missed between 8 and 24 h of work to care for their children with uncomplicated measles.
- In 2002–2003, the direct cost of measles incurred by the Italian national health service was between €17.6 million and €22 million. This would have paid for vaccinating up to 1.9 million children, which would also have prevented many cases of mumps and rubella. The 5,154 hospitalisations during this period costed about €8.8 million (18).
- In the United Kingdom, reduced MMR vaccination uptake resulted in 2,514 cases of measles being reported in 2008/2009, which was 2,366 more than in 1998/1999 (20). The cost of treating these extra cases was estimated at £587,500 over the 2-year period. These calculations were based on estimated average cost per measles case in industrialised countries and the number of measles cases reported by Public Health England (20, 21).

**Table 1:** Number of reported cases of vaccine-preventable diseases in the European region

	1980	2011	2012	2013
Measles	851,849	37,421	37,073	26,982
Rubella	No data	621,039	9,672	30,509

From Ref. (11).

Suboptimal vaccine coverage can therefore lead to disease outbreaks that can be costly, not only for healthcare resources but also for societal considerations. They affect not only individuals but also the society as a whole. In the current age of information, even a small outbreak can have an impact on the confidence of businesses, families, and the wider society, leading to a decrease in investments, tourism, and consumption (22).

## Suboptimal economic evaluations

Pricing and reimbursement decisions for pharmaceutical products are partly based on cost-effectiveness criteria. Cost-effectiveness analysis is a very useful tool for decision-makers to help prioritising access to health technologies while taking into consideration budget constraints. Cost-effectiveness is generally considered from the healthcare provider's perspective, so as to optimise national healthcare budgets. This methodology has been widely used for vaccines assessment but is now considered to be too narrow, given that vaccines benefits are population-wide, cross-sectorial, and go beyond their direct impact on healthcare systems. Results from research are increasingly providing evidence on the wider value of vaccination as discussed in two other articles in this special issue (23–27).

**Fig. 1:**
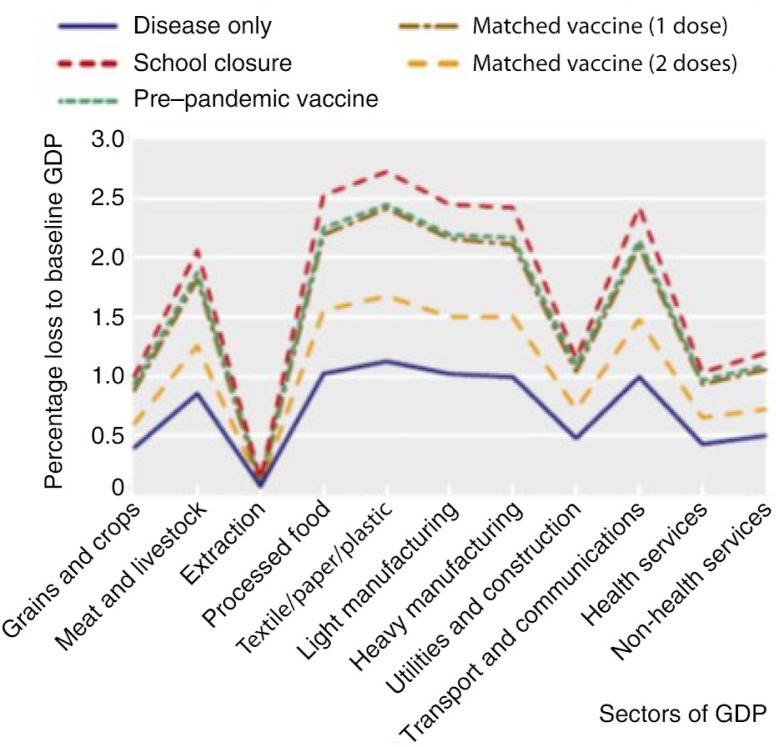
Impact of pandemic influenza on different sectors of UK gross domestic product (GDP) (28).

Although there is consensus that vaccines are vastly cost-effective, it does not seem sufficient to attract more public and private investments. However, the whole economy of a country can be impacted in the event of an outbreak of serious infectious diseases, such as pandemic influenza. It has been estimated that the largest impact of a pandemic influenza in the United Kingdom would be in the sectors of meat and livestock, processed foods, textiles, manufacturing, transport and communications, with a loss of up to 2.5% of the gross domestic product (GDP) (Fig. 1) (28). In addition to the preventable disease and healthcare costs, the preventable GDP loss needs to be taken into consideration. Potential means have been identified to increase incentives to invest in vaccines and to account more accurately for the full economic value of vaccines. In particular, Health Authorities could use a long-term horizon and a lower discount rate for vaccines than for drugs when evaluating health technologies or assign a higher cost-effectiveness threshold to take into consideration some of their intangible value (1). Indeed, it has been estimated that if ‘intangible’ values were to be included in a cost–benefit analysis, the cost–benefit ratio would be improved by a factor from 10 to 100, thus providing a rationale to invest in vaccine development (29). Not taking the full economic return from vaccines into consideration can therefore result in an underestimation of their cost-effectiveness and may delay the access of citizens to effective preventive measures.

## Suboptimal procurement processes

Vaccines are highly technical and complicated products requiring a lengthy and expensive manufacturing process with specialised handling, quality control, and procurement procedures. Their biological nature means that each production cycle corresponds to the manufacturing of a new vaccine which leads to major challenges in terms of reproducibility and consistency and to a high level of uncertainty at any time during the process. Consequently, only few players have developed the expertise to deliver to a market with a steadily increasing demand. Cost-containment efforts in European countries and procurement through tenders awarded based solely on lowest price can also threaten the short- and long-term ability of the European vaccines industry to meet the vaccine needs of countries in the European Union. Shortages of vaccines have occurred in the past and still continue to occur today. The reasons for these shortages are multi-factorial, including some companies leaving the vaccine market place because they consider it is no longer profitable, manufacturing issues or difficulties in stockpiling due to expiry date, and cold chain management. These shortages can affect millions of at-risk individuals, such as infants, and can lead to delay of certain vaccinations, leading to an increased risk for vaccine-preventable disease. The costs associated with shortages are thought to be high. Using a model developed by the CDC and applied to pertussis in the United States in 2007, in the absence of stockpile, shortage could lead to 53 pertussis hospitalisations in the best case scenario (i.e., 10% shortage and low incidence of pertussis) and up to 4,183 hospitalisations in the worst case scenario (i.e., 40% shortage and high incidence of pertussis) (30). The cost of hospitalisation for pertussis was estimated to be $6,577 in 2007 for an infant (31). This resulted in an estimated cost ranging from $350,000 to $27 million, depending on the level of dose shortage, that is, 10–40%, due to insufficient stockpiles (30).

The 2011–2012 influenza season was marked by a substantial shortage of seasonal influenza vaccine in several European regions due to anomalies identified in certain vaccine batches. The shortages most notably affected countries where procurement, either nationally or in specific regions, was sourced from a single supplier, such as in Spain, Germany, and Italy (32, 33).

These procurement practices can therefore increase the risk of vaccine shortages by discouraging market entry into countries where the price does not provide long-term industry incentives in quality and innovation, and by limiting the options to approach other suppliers should those who are contracted experience production difficulties.

## Low prices can result in inefficient long-term economic dynamics

Current public procurement systems for vaccines are essentially managed by budget holders whose objective is to obtain the best quality product at the lowest possible price to afford the quantities necessary to cover large populations. They usually do not consider the macro-economic income of the vaccine industry within the global economy. In the longer term, low prices can lead to perverse economic dynamics as they do not provide adequate resources to be invested in improving vaccine technology and expanding disease prevention through research and development. For example, over the past 3 years, the average selling price of trivalent influenza vaccines sold in bulk in Europe has fallen by half to the current average selling price of around €3.50 per dose (34). Cost-containment and tender processes, based solely on the criteria of the lowest price, are detrimental to manufacturers who, in the long run, will not obtain the necessary return on investment to remain in the market and may reduce investment in research, thus leading to reduced innovation in this sector.

The situation of under-priced vaccines in Western countries has previously resulted in a critical procurement situation with unprecedented impact for vaccine supply in developing countries. Between 1998 and 2001, 70% of UNICEF suppliers stopped part or all of their paediatric vaccine production due to lack of business profitability. With only two manufacturers left on the market, vaccination programmes were placed at significant risk in terms of supply capacity but also in terms of price stability in the absence of adequate competition. It is only recently that manufacturers have returned following the implementation of a procurement strategy by GAVI spanning several years which thus results in a more sustainable marketplace in developing countries (35).

This illustrates that the differences between vaccines and drugs in terms of complexity and production costs are not fully understood. In comparison with drugs, vaccines are highly complex products produced in extensively regulated facilities. Research on production technology is needed to bring down production costs. The same is true for development of new vaccines, new combination vaccines, and new delivery systems. These funds are not available when prices do not support resources for research and development (36). As a result, there will be increasingly fewer players on the market place, which will impact the public health and the socio-economic fabric of the country.

## Conclusion

The combined effect of mistrust of the population in vaccination, the suboptimal economic evaluation methods that only account for the healthcare-related benefits of vaccines, and the lack of recognition by governments and other purchasers of the complexity of vaccine manufacturing process can result in no wealth creation. This is exactly the opposite of what is expected from preventive therapies such as vaccines that are developed to maintain population health, and health is wealth. Indeed, if payers do not see all the benefits from vaccination, but only the costs, they will underinvest. If doctors do not see diseases prevented by vaccination, but only the time spent in monitoring vaccination programmes, they will not understand the importance in their practice. If parents no longer see children sick with serious infectious diseases, but hear only about the ‘dangers’ of vaccination, they will be less willing to have their healthy children vaccinated. Therefore, adequate support and communication from health authorities and governments is necessary to promote the benefits of vaccination and reduce the risk of outbreaks of infectious diseases that are a threat for national public health and economy.

More incentives for the development of vaccines could also contribute to building a healthier society, instead of a society of chronically sick people, by assigning more economic value to disease prevention (29). A number of measures to make vaccines more attractive to industry have been identified. For example, vaccine development could benefit from tax credits and public–private partnerships, and vaccines’ specificities could be taken into consideration in economic evaluations. While cost-effectiveness analyses are useful to inform decision-makers about the efficiency of vaccines, they should also be coupled with other economic evaluations accounting for the broader impact of vaccination on the society and the economy as a whole and estimating the return on investment of vaccination programmes to inform about their affordability.

In conclusion, governments, international agencies, and other stakeholders, such as the medical community, have an important role to play to help society regain confidence in vaccination and ensure that the full benefits of vaccination programmes are fully recognised and valued.
